# Effects of Coronavirus Fears on Anxiety and Depressive Disorder Symptoms in Clinical and Subclinical Adolescents: The Role of Negative Affect, Intolerance of Uncertainty, and Emotion Regulation Strategies

**DOI:** 10.3389/fpsyg.2021.716528

**Published:** 2021-08-06

**Authors:** Bonifacio Sandín, Victoria Espinosa, Rosa M. Valiente, Julia García-Escalera, Julia C. Schmitt, Sandra Arnáez, Paloma Chorot

**Affiliations:** Facultad de Psicología, Universidad Nacional de Educación a Distancia, Madrid, Spain

**Keywords:** coronavirus fears, transdiagnostic, emotion regulation, anxiety, depression, COVID-19, negative affect, intolerance of uncertainty

## Abstract

Fears related to COVID-19 (“coronavirus fears”) have emerged as a new psychological effect of the current COVID-19 pandemic and have been associated with psychological distress and impairment. Other adverse effects include an increase in anxiety and depression symptoms and the respective disorders. The purpose of the current study was to examine the incremental validity of coronavirus fears and transdiagnostic factors in the prediction of the severity of anxiety and depressive disorder symptoms. A sample of 144 adolescents [aged 12–18 years, 55 boys (38.2%) and 89 girls (61.8%)] most of whom showed elevated levels of anxiety and depressive disorder symptoms completed several self-report measures online assessing coronavirus fears, transdiagnostic vulnerability and protective factors, and emotion regulation strategies. Results based on a series of hierarchical multiple regression analyses revealed that coronavirus fears, negative affect, intolerance of uncertainty, acceptance/tolerance, rumination and suppression explained unique variance in the severity of anxiety and depressive disorder symptoms. Path analysis demonstrated that acceptance/tolerance, rumination and suppression mediated the association between higher level transdiagnostic factors and the severity of major depressive disorder symptoms. Findings provide support for the hierarchical transdiagnostic model of emotional disorders and suggest that clinicians should be aware of coronavirus fears. Also, the results warrant the need to consider transdiagnostic vulnerability and protective processes in the new protocols for the treatment of emotional disorders.

## Introduction

Anxiety and depressive symptoms and disorders are very common mental health problems in adolescents. A meta-analysis of 41 studies conducted in 27 countries estimated a worldwide pooled prevalence in children and adolescents of any anxiety disorder of 6.5% and of any depressive disorder of 2.6% (Polanczyk et al., 2015). According to this study, the highest prevalence rate was found for anxiety disorders, followed by disruptive disorders (5.7%) and attention-deficit/hyperactivity disorder (3.4%). In a recent literature review, Sandín et al. (2018) found that prevalence estimates of anxiety in children and adolescents vary significantly across studies, ranging from 8.3 to 32.4% for any anxiety disorder. Several factors, including the criteria for selecting participants, diagnostic procedures, and the definition of functional impairment could explain the high variability. Subclinical symptoms of anxiety and depression are also very prevalent, having been estimated to be present in 32 and 29.2% of adolescents, respectively; likewise, these symptoms have been related to functional impairment and suicidality (Balázs et al., 2013).

In addition to the high prevalence of these disorders, anxiety and depression overlap across the life span, existing high comorbidity in children and adolescents. It has been reported that 25–50% of depressed youth have anxiety disorders and 10–15% of anxious youth have depression (Axelson and Birmaher, 2001), with comorbidity rates as high as 75% in clinical samples (Balázs et al., 2013). Children and adolescents with anxiety and/or mood disorders also share a number of vulnerability factors, including temperament (behavioral inhibition, neuroticism or negative affect) and maladaptive emotion regulation strategies that in the long run maintain anxiety and depression symptoms (Ehrenreich-May et al., 2018). The conceptual overlap between anxiety and depressive disorders, the common clinical features (overestimation of threat, shared symptoms, etc.), the commonalities in cognitive, behavioral and emotional facets of dysregulation (selective attention to threat, expectancy biases, etc.), and the shared general biological vulnerability (negative affect or neuroticism), suggest that a “transdiagnostic” approach could be more appropriate than a disorder-specific perspective to understand and manage these disorders (Sandín et al., 2012a; García-Escalera et al., 2016). A transdiagnostic process has been defined as “a major factor that can explain the maintenance of numerous disorders that an individual may experience” (Egan et al., 2012, p. 280). The transdiagnostic approach is a new focus in clinical psychology that formalizes mental disorders based on a set of etiological processes or factors, cognitive and behavioral, that are shared by groups of mental disorders, e.g., emotional disorders (Sandín et al., 2020a).

In the last few years many studies have highlighted the role of transdiagnostic constructs as common etiopathogenic factors of emotional disorders, especially of anxiety and depressive disorders, including, for example, positive and negative affect (Clark and Watson, 1991), neuroticism (Barlow et al., 2014), anxiety sensitivity (Taylor, 1999), distress tolerance (Sandín et al., 2017), emotion regulation strategies (Aldao, 2012; Ferrer et al., 2018), intolerance of uncertainty (Einstein, 2014; Pineda, 2018), emotional avoidance (Ehrenreich-May et al., 2018), and perfectionism (Egan et al., 2012; see Sandín et al., 2012a and Barlow et al., 2014, for reviews of potential transdiagnostic constructs.). The PANAS has been used extensively to assess the temperamental dimensions. In a first modern conceptualization of temperament related to anxiety and depression, Clark and Watson (1991) stated two main genetically based temperamental dimensions, i.e., negative affect or neuroticism and positive affect or extraversion, and proposed the well-known tripartite theory of anxiety and depression. According to this model, general distress (negative affect) is a common temperamental factor for anxiety and depression, while anhedonia (low positive affect) is specific for depression (e.g., see Watson et al., 2008). The temperamental concept of negative affect has been used as equivalent to the concept of neuroticism (Barlow et al., 2014) and is currently integrated as a temperamental dimension for internalizing disorders in the HiTOP hierarchical taxonomy of psychopathology (Kotov et al., 2021).

Recently, Sandín et al. (2020a) developed a hierarchical transdiagnostic model of emotional disorders. It consists of a hierarchy of causal transdiagnostic factors of emotional disorders that represent different levels of commonality. The highest level of the model describes more general transdiagnostic factors, i.e., factors of general vulnerability, which represent temperament and include behavioral inhibition, neuroticism, negative affect, and positive affect. Lower levels of the model include clinical traits (e.g., intolerance of uncertainty, distress tolerance, anxiety sensitivity, and perfectionism) and coping strategies (e.g., reappraisal, suppression, acceptance, and cognitive avoidance). The model states that people with high levels of negative affect or neuroticism tend to experience intense levels of negative emotions in stressful situations (first level); thus, individuals with high levels of negative affect tend to react to pandemic-related stress with intense emotional distress. Depending on certain clinical traits (second level), such people may experience negative reactions to these emotions, due to, for example, high levels of anxiety sensitivity (fear of anxiety symptoms due to the belief that such symptoms are dangerous) or high intolerance of uncertainty (negative reactions to unpredictable negative events). The individual can try to alleviate or manage their distress by reacting with various emotion regulation or coping strategies (third level), such as avoidance, rumination, suppression, reappraisal, acceptance, etc.

Emotion regulation strategies can be adaptive or maladaptive (e.g., Aldao et al., 2010). Adaptive strategies are associated with LESS psychopathology and maladaptive strategies with MORE psychopathology. Thus, while acceptance, awareness, reappraisal, and self-instructions have been associated with less psychopathology (adaptive strategies), rumination, suppression, and distraction have been related to more psychopathology (maladaptive strategies) (Aldao et al., 2010; Ehrenreich-May et al., 2018). Therefore, we may expect a negative association of anxiety and depression with adaptive strategies, and a positive association with maladaptive strategies.

The transdiagnostic approach to emotional disorders could be an appropriate way to investigate common anxiety and depression in situations of high psychosocial stress, such as the current crisis generated by the COVID-19 pandemic. In this regard, we believe that the transdiagnostic model of emotional disorders could provide an appropriate framework for examining the effect of different kinds of transdiagnostic variables on anxiety and depressive symptomatology. These types of variables could correspond to different levels of the model, including the levels of general vulnerability, clinical traits, and coping strategies. Likewise, and in line with preliminary studies reported by Lee's group (Lee and Crunk, 2020; Lee et al., 2020), an increase in anxiety and depressive symptoms associated with coronavirus fears could be expected in such a way that coronavirus fears could have an incremental effect on the outcome measures, above the effect of the transdiagnostic variables specified in the transdiagnostic model.

The current COVID-19 pandemic has a serious impact on people's health around the world. The bulk of evidence suggests that individuals of the general population who were kept in isolation and quarantine experienced significant stress and emotional impact, showing relatively high rates of anxiety and depression symptoms. A recent meta-analysis reported by Salari et al. (2020) based on 17 studies of the general population found that the prevalence of anxiety and depression, as a result of the COVID-19 pandemic, was 31.9 and 33.7%, respectively. Similar results have been found in studies of the general population conducted in Spain (Hidalgo et al., 2020; Sandín et al., 2020c, 2021; Gutiérrez-Hernández et al., 2021) and Hispanic American countries (Andrades-Tobar et al., 2021; Mestas et al., 2021) since the COVID-19 pandemic, as well as in children and adolescents (Gómez-Becerra et al., 2020; Orgilés et al., 2020b, 2021; Pedreira, 2020). The existing literature on the impact of COVID-19 in adolescents is generally limited, and evidences the relevance of researching this issue (e.g., Muzi et al., 2021), including the role of emotion regulation strategies (Velotti et al., 2021).

Fear is a primitive alarm response to present danger and is related to action, particularly to escape and avoidance. However, when the action is blocked or thwarted, for example because the danger is uncontrollable, fear turns into anxiety (Öhman, 1993). As this author suggested, fear often develops into anxiety when attempts to cope with a threat are unsuccessful. In addition, along with the often observed finding that anxiety tends to precede the occurrence of depression, it has been reported that at least certain types of depression are complications of anxiety occurring in some people under certain conditions. Stressful negative life events can lead to clinical anxiety and, possibly some time later, to depression (Barlow, 2002). It has been stated that fears during developmental stages (i.e., during childhood and adolescence) can be a risk factor for the development of anxiety disorders and other emotional disorders (Sandín, 1997).

Research conducted in Spain during the mandatory national quarantine revealed that individuals exposed to the pandemic experienced coronavirus fears very frequently (Sandín et al., 2020c). In this study we found that the most common fears mainly concerned fears related to infection, disease and death on account of COVID-19, and fears related to work and social isolation. Several of these fears (rated as “much” or “extremely”) were found in nearly half of the studied general population sample (in more than 40%). In general, one out of four participants suffered from coronavirus fears, being more prevalent in women than in men. Other authors also reported that coronavirus fears were among the primary emotional responses to the pandemic (Khattak et al., 2020; Teng et al., 2021).

Prior studies based on other viral epidemics have reported that people tend to experience fears of infection, which result in increased anxiety and depression (Hall et al., 2008). Fear is an automatic emotion that occurs in response to awareness of a threat (Öhman, 1993; Barlow, 2002), and is one of the major underlying factors that can lead to mental health issues (Kumar and Nayar, 2020; Teng et al., 2021). Although research is very preliminary, coronavirus fears have been associated with elevated depression, generalized anxiety, and death anxiety (Lee and Crunk, 2020; Lee et al., 2020; Yildirim et al., 2021). All these authors demonstrated that coronaphobia predicted pandemic-related anxiety and depression in adults from the general population. Thus, a new line of research is related to the notion that fear is a major contributing factor in the elevated rates of anxiety and depression during the COVID-19 pandemic. It has been suggested that emotional responses related to COVID-19, including increased anxiety and depression, may result from increased fear of coronavirus (Harper et al., 2020; Lin, 2020).

However, no study has yet systematically examined the effect of coronavirus fears on anxiety and depressive disorder symptom severity during this pandemic (for example, Lee et al., 2020 assessed anxiety and depression using only two screening items for each). On the other hand, the current bulk of evidence on this issue is based on studies carried out with adults from the general population. To date, the extent to which coronavirus fears are responsible for the severity of anxiety and depression that is being observed in adolescents during the COVID-19 pandemic has not yet been investigated. Given the lack of previous investigations on these issues, a first purpose of the present study was to examine the unique contribution of coronavirus fears to the prediction of anxiety and depressive disorder symptom severity in adolescents. Some transdiagnostic factors (e.g., affectivity, intolerance of uncertainty, distress tolerance and emotion regulation strategies) have been etiologically implicated in emotional disorders (Barlow et al., 2014; Pineda, 2018; Sandín et al., 2020a). Thus, the first primary hypothesis of the present study was that coronavirus fears should predict anxiety and/or depressive disorder symptoms severity beyond relevant transdiagnostic factors. We expected an incremental predictive effect of coronavirus fears on the outcome measures above the possible predictive effect of positive and negative affect, intolerance of uncertainty, distress tolerance and emotion regulation strategies.

As described above, the transdiagnostic approach provides a theoretical framework to examine the impact of the COVID-19 pandemic on anxiety and depressive symptomatology. Some studies (Lee and Crunk, 2020; Lee et al., 2020) have reported preliminary information concerning a possible role of individual difference variables (neuroticism, health anxiety, and reassurance-seeking) in the prediction of anxiety and depression during the COVID-19 pandemic in adults. However, no study has yet systematically examined the predictive association between main transdiagnostic factors and the severity of anxiety and depressive symptomatology. This problem has not yet been investigated within the adolescent population either. Therefore, a second aim of this study was to preliminarily test the validity of the hierarchical transdiagnostic model of emotional disorders developed by Sandín et al. (2020a). According to this model, we expected that variables pertaining to the first three levels of the model (i.e., general factors, clinical traits, and emotion regulation strategies) should make a unique contribution to explaining variance in anxiety and depressive disorder symptom severity.

## Method

### Participants

The sample consisted of 144 adolescents, most of whom showed elevated levels of anxiety and/or depressive symptoms (76.4%) and some of whom (34%) met the diagnostic criteria for an anxiety disorder or a major depressive disorder; 53.1% of these clinical adolescents met the diagnostic criteria for one or more comorbid anxiety or depressive disorder. The mean age of the sample was 14.6 years (range: 12–18 years; *SD* = 1.9). There were 55 boys (38.2%) and 89 girls (61.8%). All adolescents were Spanish residents. Other demographic characteristics of the sample are shown in Table 1.

**Table 1:** Sociodemographic characteristics of the sample (*N* = 144).

Age (years, mean/*SD*)	14.6 (1.9)
Gender (*n*/%)
Boys	55 (38.2)
Girls	89 (61.8)
Adolescent guardian^a^
Mother	122 (84.7)
Father	20 (13.9)
Other	2 (1.4)
Family income level (yearly, *n*/%)
Up to 10.000 €	20 (13.9)
10,000–25,000 €	85 (59.0)
25,000–40,000 €	16 (11.1)
More than 40,000 €	1 (7.6)
No information	12 (8.3)
Family life
Lives with both parents	103 (75.1)
Lives with mother	28 (19.0)
Lives with father	2 (1.4)
Lives with other family members	11 (7.6)
Country of birth (*n*/%)
Spain	129 (89.6)
Other countries	15 (10.4)
Country of birth of the guardian (*n*/%)
Spain	114 (79.2)
Other countries	30 (20.8)
Marital status of the guardian (*n*/%)
Married	104 (72.2)
Single/never married	12 (8.3)
Cohabitating (with partner)	12 (8.3)
Separated	16 (11.1)
Education level of the guardian (*n*/%)
College	34 (23.6)
High school	56 (38.9)
Less than high school	53 (36.8)
Space of the house (*n*/%)
<50 m^2^	2 (1.4)
50–90 m^2^	32 (22.2)
>90 m^2^	110 (76.4)

a *Contact family member for assessment*.

### Measures

*Revised Child Anxiety and Depression Scale*−*30* (RCADS-30; Sandín et al., 2010). The RCADS-30 is a 30-item self-report scale that comprises the following subscales derived from the Diagnostic and Statistical Manual of Mental Disorders (DSM-IV/5) criteria (5 items per subscale): (1) social phobia (SP), (2) generalized anxiety disorder (GAD), (3) panic disorder (PD), (4) separation anxiety disorder (SAD), (4) obsessive-compulsive disorder (OCD), and (6) major depressive disorder (MDD). This scale has previously demonstrated good psychometric properties (Piqueras et al., 2017). Each item is scored from 0 (“Never”) to 3 (“Always”), with higher scores representing more severe symptoms. In the current sample, the alpha coefficients of the RCADS-30 were as follows: RCADS-30-Total score (α = 0.92), RCADS-30-Anxiety (α = 0.90) and RCADS-30-MDD (α = 0.80). The RCADS-30 is an overall measure of anxiety, depression and OCD symptoms. To estimate the RCADS-30-Anxiety score, OCD and MDD subscales were deleted in order to obtain a specific measure of anxiety disorder symptoms.

*Coronavirus Fears Scale [Escala de Miedos al Coronavirus]* (EMC; Sandín et al., 2020c). The EMC includes 18 items related to fears concerning the psychosocial aspects of COVID-19, such as the fear that some relative gets the virus or the fear related to social isolation. The scale was adapted for the adolescent population. The version of the EMC for adolescents includes the same 18 items than the original scale. All of the original items were revised by the authors in order to adapt them to the adolescent population. The reviewers provided alternatives for some items related to both content and wording. For example, the original Item 6 “That you could lose your job or part of your job” was transformed into “That a close relative loses the job.” All authors agreed to the final draft. Items can be rated using an intensity scale of five points, ranging from 1 (“Not at all or very little”) to 5 (“Very much or extremely”). The scale demonstrated excellent internal consistency reliability (α = 0.93) within the present sample.

*Positive and Negative Affect Schedule for Children and Adolescents [Escalas PANAS de Afecto Positivo y Negativo para Niños y Adolescentes]* (PANASN; Sandín, 2003). The PANASN provides scores for 2 subscales of 10 items each, measuring positive and negative affect. Participants are asked to rate items according to how they usually feel on a scale from 1 (“Never or almost never”) to 3 (“A lot of the time”). This self-report questionnaire has demonstrated adequate psychometric properties (Sandín, 2003). In the present study, we found a reliability of α = 0.77 for positive affect and α = 0.82 for negative affect.

*Intolerance of Uncertainty Scale*−*12* (IUS-12; Carleton et al., 2007). We used the Spanish version by Sandín et al. (2012b). The IUS-12 is a self-report scale which comprises 12 items that assess intolerance of ambiguous situations and uncertainty of future events. Items can be rated on a scale ranging from 1 (“Not characteristic of me”) to 5 (“Totally characteristic of me”). Evidence has been provided on its excellent psychometric properties (Pineda, 2018). In the present study, its coefficient alpha was 0.87.

*Distress Tolerance Scale* (DTS; Simons and Gaher, 2005). We used the Spanish version of the scale (Sandín et al., 2017). The DTS is a 15-item self-report scale designed to assess the degree to which individuals experience and withstand distressing psychological states. Participants rate the items on a 5-point scale ranging from 1 (“Strongly agree”) to 5 (“Strongly disagree”). Higher scores indicate a greater ability to tolerate emotional distress. The measure demonstrated excellent internal consistency reliability (α = 0.91) within the present sample.

*Emotion Regulation Strategies Questionnaire [Cuestionario de Estrategias de Regulación Emocional]* (CERE; Sandín et al., 2008). The CERE was designed to assess different emotion regulation strategies. It includes the following seven subscales (find number of items and alpha coefficients within the present sample in parentheses): (1) Awareness and understanding emotions (6 items; α = 0.82), (2) Acceptance and tolerance (6 items; α = 0.76), (3) Reappraisal (4 items; α = 0.67), (4) Self-instructions (3 items; α = 0.82), (5) Suppression (3 items; α = 0.77), (6) Rumination (3 items; α = 0.62), and (7) Distraction (3 items; α = 0.73).

*Mini International Neuropsychiatric Interview for Children and Adolescents* (MINI-KID; Sheehan et al., 1998). The MINI-KID is a structured diagnostic interview for individuals aged from 6 to 17 years. It is based on the DSM-IV and ICD-10 criteria for psychiatric disorders. The reliability and validity of the MINI-KID has been demonstrated (Sheehan et al., 2010).

### Procedure

The subjects were adolescents selected to participate in an internet-delivered version of the Unified Protocol for Transdiagnostic Treatment of Emotional Disorders in Adolescents (iUP-A; Sandín et al., 2019, 2020b) who were recruited through school counselors' referrals from four secondary schools in Castilla–La Mancha and Madrid (Spain). In order to be able to participate in the study, adolescents had to be between 12 and 18 years old, reside in Spain and have access to a computer or tablet. We assessed the following exclusion criteria through telephone calls and online questionnaires: (a) having been diagnosed with a severe psychopathology such as psychotic disorder, bipolar disorder, severe depressive disorder, intellectual disability, severe learning disability, autism spectrum disorder or substance dependence, or an illness incompatible with the participation in the program; (b) being at moderate or severe risk for suicide; (c) currently receiving psychological treatment; (d) having changed the medication dosage for the treatment of o psychological or psychiatric problem in the last 3 months; or (e) not having given informed consent.

The adolescents completed the self-report questionnaires online. The PANASN and DTS were completed by 99 participants (in the regression analyses, missing values were replaced by the mean). Those adolescents who scored above the clinical cut-off on one or more of the RCADS-30 subscales (Piqueras et al., 2017) were invited to attend the MINI-KID. The MINI-KID was conducted separately with each adolescent and their guardian via video call. The informed consent was signed by the adolescent and their parents (when the adolescent was under 16 years old), and was returned via email. Information concerning anonymity and privacy was delivered in the informed consent. Ethical approval was granted by the Research Ethics Committee of the Universidad Nacional de Educación a Distancia. No incentives were provided to the adolescents or their parents for participating in the present study.

### Statistical Analysis

Apart from basic statistics (means and standard deviations), we estimated the coefficient alpha (α) to examine the reliability (internal consistency) of the instruments. Normality of the variables was assessed by means of Kolmogorov-Smirnov tests of goodness of fit. Data provided no evidence against the null hypothesis that the sample had been drawn from a normal population. D ranged from 0.052 (exact *p* = 0.941) to 0.132 (exact *p* = 0.059). In addition, before calculating the regression analyses we checked for the issue of multicollinearity between the predictors. Values of the variance inflation factor (VIF) were <10 (VIFs ranged from 1.33 to 3.14), thus it appears that multicollinearity was not a threat to the validity of the regression analyses. The correlations between variables were calculated by means of Pearson product-moment correlations.

Three separated hierarchical multiple linear regression analyses were carried out to examine the unique contribution of general personality factors, clinical traits, emotion regulation strategies and coronavirus fears to the prediction of the three outcome measures (combined anxiety and depression, anxiety, and depression). The hierarchical multiple regression analyses were performed to assess incremental validity. The predictor variables were included in the equation in four separate blocks in order to differentiate the effects of each transdiagnostic level (first three blocks) and to examine the incremental validity of coronavirus fears (fourth block). Before calculating the hierarchical regression analyses, three preliminary multiple regression analyses were conducted for each of the three outcome measures including all the sociodemographic variables as predictors. The sociodemographic variables (categorical variables) were recoded into dummy variables (all sociodemographic variables except age). Finally, a series of path analyses was carried out to examine the mediation hypothesis, conducting parallel multiple mediation analyses, using ordinary least squares path analysis. A bias-corrected bootstrapping sampling procedure based on 10,000 bootstrap samples was applied to assess indirect effects. Descriptive statistics and regression analyses were computed with the statistical software IBM SPSS Statistics 24.0. Mediation analyses were conducted using the PROCESS macro for SPSS (Hayes, 2013).

## Results

### Descriptive Statistics and Correlations

Descriptive statistics (means and standard deviations) and alpha coefficients of the measures used in the present study are shown in Table 2. Zero-order correlations demonstrated that coronavirus fears were significantly related to anxiety disorder symptoms but not to depressive disorder symptoms (see Table 2). Negative affect and intolerance of uncertainty were significantly associated with all anxiety and depression variables, while distress tolerance was negatively associated. Positive affect was significantly related only to depression. Regarding the emotion regulation strategies, all of them correlated significantly with anxiety and depressive disorder symptoms, except self-instructions; thus, this last variable was not included in the regression analyses.

**Table 2:** Product-moment correlations of coronavirus fears and transdiagnostic measures with symptoms of anxiety and depressive disorders.

**Measure**	**Zero-order correlation**			
	**RCADS-30**				
	**Total score**	**Total anxiety**	**Depression**	**Mean *(SD)***	**Alpha**
*C*oronavirus fears	0.29^***^	0.36^***^	0.02	49.8 (14.7)	0.93
Negative affect	0.77^***^	0.73^***^	0.62^***^	17.8 (4.2)	0.82
Positive affect	−0.13	−0.07	−0.34^**^	22.8 (3.6)	0.77
Intolerance of uncertainty	0.67^***^	0.62^***^	0.53^***^	30.1 (9.6)	0.87
Distress tolerance	−0.64^***^	−0.63^***^	−0.51^***^	45.8 (13.1)	0.91
Awareness/understanding	−0.37^***^	−0.35^***^	−0.37^***^	17.8 (5.1)	0.82
Acceptance/tolerance	−0.44^***^	−0.44^***^	−0.39^***^	17.0 (4.9)	0.76
Reappraisal	−0.22^**^	−0.21^*^	−0.20^*^	12.4 (3.4)	0.67
Self-instructions	−0.02	−0.02	−0.10	9.5 (3.2)	0.82
Suppression	0.32^***^	0.23^**^	0.42^***^	9.8 (3.4)	0.77
Rumination	0.58^***^	0.51^***^	0.50^***^	7.6 (2.8)	0.62
Distraction	0.22^*^	0.17^*^	0.20^*^	9.0 (2.9)	0.73
Mean *(SD)*	27.4 (14.2)	18.1 (9.6)	5.1 (3.2)		
Alpha	0.92	0.90	0.80		

* *p < 0.05,*

** *p < 0.01,*

*** *p < 0.001*.

### Prediction of Anxiety and Depressive Disorder Symptoms: Hierarchical Multiple Regression Analyses

A series of hierarchical multiple linear regression analyses was performed to examine the relationship of coronavirus fears and transdiagnostic variables with the three outcome variables (RCADS-30-Total score, RCADS-30-Anxiety and RCADS-30-MDD). Three preliminary stepwise selection multiple regression analyses were conducted for each of the three outcome measures including all the sociodemographic variables as predictors (see Table 1). None of these multiple regression analyses were statistically significant, i.e., no independent variable significantly predicted the outcome variable when all predictors were included in the model; RCADS-30-Total score, *R*^2^ = 0.13, *F*_(18, 112)_ = 0.93 ns; RCADS-30-Anxiety, *R*^2^ = 0.14, *F*_(18, 112)_ = 1.0 ns; RCADS-30-MDD, *R*^2^ = 0.11, *F*_(18, 112)_ = 0.77 ns. Thus, sociodemographic variables were not included in successive analyses.

Three separate hierarchical multiple regression analyses with one of the three outcome variables as the dependent variable were carried out to assess incremental validity. The first step of each regression included the two general personality factors (positive and negative affect). In the second step we added the two maladaptive (clinical) traits, i.e., intolerance of uncertainty and distress tolerance. In the third step we added the emotion regulation strategies, which are awareness and understanding emotions, acceptance and tolerance, reappraisal, suppression, rumination, and distraction. In the final step we added the variable coronavirus fears (see Tables 3, 4 for regression summaries).

**Table 3:** Hierarchical multiple regression analyses examining the role of transdiagnostic measures and coronavirus fears (incremental validity) in the prediction of anxiety and depressive disorder symptoms.

	**RCADS-30-Total score**		**RCADS-30-Anxiety** ^**a**^		**RCADS-30-MDD** ^**b**^	
**Predictors added at each step**	***R^**2**^***	***ΔR^**2**^***	***R^**2**^***	***ΔR^**2**^***	***R^**2**^***	***ΔR^**2**^***
Step 1 (general personality traits)^c^	0.40^***^	0.40^***^	0.35^***^	0.35^***^	0.29^***^	0.29^***^
Step 2 (clinical traits)^d^	0.56^***^	0.16^***^	0.49^***^	0.14^***^	0.40^***^	0.11^***^
Step 3 (emotion regulation strategies)^e^	0.66^***^	0.10^***^	0.58^***^	0.09^**^	0.53^***^	0.13^***^
Step 4 (coronavirus fears)	0.71^***^	0.05^***^	0.65^***^	0.07^***^	0.54^***^	0.01

*R^2^ and ΔR^2^ for each step.*

a *The OCD and MDD subscales of the RCADS-30 were deleted.*

b *Major Depressive Disorder subscale.*

c *Negative and positive affect.*

d *Intolerance of uncertainty, and Distress tolerance.*

e *Awareness/Understanding, Acceptance/Tolerance, Reappraisal, Self-instructions, Suppression, Rumination, and Distraction.*

** *p < 0.01,*

*** *p < 0.001*.

**Table 4:** Hierarchical multiple regression analyses examining the role of transdiagnostic measures and coronavirus fears in the prediction of anxiety and depressive disorder symptoms.

**Predictors**	**RCADS-30-Total score**			**RCADS-30-Anxiety** ^**a**^			**RCADS-30-MDD** ^**b**^		
	***B***	***SE B***	**β**	***B***	***SE B***	**β**	***B***	***SE B***	**β**
General personality traits:
Positive affect	0.23	0.25	0.05	0.19	0.18	0.06	−0.10	0.07	−0.10
Negative affect	1.37	0.30	0.34^***^	0.89	0.22	0.32^***^	0.15	0.08	0.17
Clinical trials:
Intolerance of uncertainty	0.43	0.09	0.29^***^	0.27	0.07	0.27^***^	0.06	0.02	0.19^*^
Distress tolerance	0.20	0.10	0.16^*^	0.12	0.07	0.13	0.02	0.03	0.06
Emotion regulation strategies:
Awareness/understanding	0.01	0.20	0	0.08	0.15	0.04	−0.05	0.06	−0.08
Acceptance/tolerance	−0.97	0.23	−0.33^***^	−0.72	0.17	−0.37^***^	−0.15	0.06	−0.23^*^
Reappraisal	−0.13	0.23	−0.03	−0.03	0.17	−0.01	−0.04	0.06	−0.04
Suppression	0.62	0.24	0.15^**^	0.27	0.18	0.10	0.22	0.07	0.24^***^
Rumination	0.97	0.30	0.19^**^	0.55	0.23	0.16^*^	0.23	0.08	0.20^**^
Distraction	0.41	0.27	0.08	0.19	0.21	0.06	0.07	0.08	0.06
Coronavirus fears	0.21	0.05	0.21^***^	0.18	0.03	0.27^***^	−0.01	0.02	−0.01

*Final step summary.*

a *The OCD and MDD subscales of the RCADS-30 were deleted.*

b *Major depressive disorder subscale.*

*B, regression coefficient; SE B, standard error of B; β, standardized regression coefficient.*

* *p < 0.05,*

** *p < 0.01,*

*** *p < 0.001*.

RCADS-30-Total score was the outcome variable in the first regression analysis. In the first step, when positive and negative affect were added, negative affect emerged as the only significant predictor (β = 0.63, *p* < 0.001), *R*^2^ = 0.40, *F*_(2, 141)_ = 47.10, *p* < 0.001. In Step 2, when intolerance of uncertainty and distress tolerance were entered, negative affect (β = 0.33, *p* < 0.001) and intolerance of uncertainty (β = 0.46, *p* < 0.001) were the only significant predictor variables, *R*^2^ = 0.56, *F*_(4, 139)_ = 44.5, *p* < 0.001. In Step 3, when emotion regulation strategies were added, negative affect (β = 0.32, *p* < 0.001), intolerance of uncertainty (β = 0.33, *p* < 0.001), acceptance/tolerance (β = −0.31, *p* < 0.001), suppression (β = 0.12, *p* < 0.05) and rumination (β = 0.20, *p* < 0.01) emerged as the only significant predictors, *R*^2^ = 0.66, *F*_(10, 133)_ = 26.7, *p* < 0.001. Finally, in Step 4, when the variable coronavirus fears was added, negative affect (β = 0.33, *p* < 0.001), intolerance of uncertainty (β = 0.29, *p* < 0.001), distress tolerance (β = −0.16, *p* < 0.05), acceptance/tolerance (β = −0.33, *p* < 0.001), suppression (β = 0.15, *p* < 0.01), rumination (β = 0.19, *p* < 0.01), and coronavirus fears (β = 0.21, *p* < 0.001) were the only significant predictors in the model, *R*^2^ = 0.71, *F*_(11, 132)_ = 29.9, *p* < 0.001.

In the second regression analysis, RCADS-30-Anxiety was the outcome variable. In Step 1, when positive and negative affect were added, negative affect (β = 0.60, *p* < 0.001) was the only significant predictor, *R*^2^ = 0.35, *F*_(2, 141)_ = 37.4, *p* < 0.001. In Step 2, when intolerance of uncertainty and distress tolerance were entered, negative affect (β = 0.29, *p* < 0.01) and intolerance of uncertainty (β = 0.41, *p* < 0.001) were the only significant predictor variables, *R*^2^ = 0.49, *F*_(4, 139)_ = 32.6, *p* < 0.001. In Step 3, when emotion regulation strategies were added, negative affect (β = 0.30, *p* < 0.001), intolerance of uncertainty (β = 0.32, *p* < 0.001), acceptance/tolerance (β = −0.33, *p* < 0.001), and rumination (β = 0.17, *p* < 0.05) emerged as the only significant predictors, *R*^2^ = 0.58, *F*_(10, 133)_ = 18.1, *p* < 0.001. Finally, in Step 4, when the variable coronavirus fears was added, negative affect (β = 0.32, *p* < 0.001), intolerance of uncertainty (β = 0.27, *p* < 0.001), acceptance/tolerance (β = −0.37, *p* < 0.001), rumination (β = 0.16, *p* < 0.05) and coronavirus fears (β = 0.27, *p* < 0.001) were the only significant predictors in the model, *R*^2^ = 0.65, *F*_(11, 132)_ = 21.7, *p* < 0.001.

In the third regression analysis, RCADS-30-MDD was the outcome variable. In Step 1, positive and negative affect were added and both, negative affect (β = 0.47, *p* < 0.001) and positive affect (β = 0.19, *p* < 0.01), were significant predictors, *R*^2^ = 0.29, *F*_(2, 141)_ = 29.1, *p* < 0.001. In Step 2, when intolerance of uncertainty and distress tolerance were entered, negative affect (β = 0.22, *p* < 0.05), positive affect (β = −0.21, *p* < 0.01), and intolerance of uncertainty (β = 0.36, *p* < 0.001), were the only significant predictor variables, *R*^2^ = 0.40, *F*_(4, 139)_ = 22.9, *p* < 0.001. In Step 3, when emotion regulation strategies were added, intolerance of uncertainty (β = 0.19, *p* < 0.05), acceptance/tolerance (β = −0.23, *p* < 0.05), suppression (β = 0.25, *p* < 0.001), and rumination (β = 0.20, *p* < 0.01) emerged as the only significant predictors, *R*^2^ = 0.34, *F*_(10, 133)_ = 14.8, *p* < 0.001. The drop in the level of predictive power of positive and negative affect was quite surprising (these variables were no longer significant after adding the emotion regulation variables). Although to a lesser extent, this phenomenon also occurred with the intolerance of uncertainty predictor. Such changes could be due to a possible mediation effect of the emotion regulation variables. That is, some emotion regulation strategies could mediate the effect of personality variables and/or intolerance of uncertainty on depressive disorder symptoms. Finally, in Step 4, when coronavirus fears was added, the same predictors were significant as in the previous step: intolerance of uncertainty (β = 0.18, *p* < 0.05), acceptance/tolerance (β = −0.23, *p* < 0.05), suppression (β = 0.24, *p* < 0.001), and rumination (β = 0.20, *p* < 0.01), *R*^2^ = 0.54, *F*_(11, 132)_ = 13.3, *p* < 0.001.

### Emotion Regulation Strategies as Mediator Variables

As indicated above, in Step 3 (third regression analysis) the predictive power of positive and negative affect and intolerance of uncertainty dropped after adding the emotion regulation strategies to the equation, being significant acceptance/tolerance, suppression and rumination. Thus, we hypothesized that these three emotion regulation strategies could mediate the effect of affectivity and intolerance of uncertainty on the RCADS-30-MDD outcome measure.

**Figure 1:**
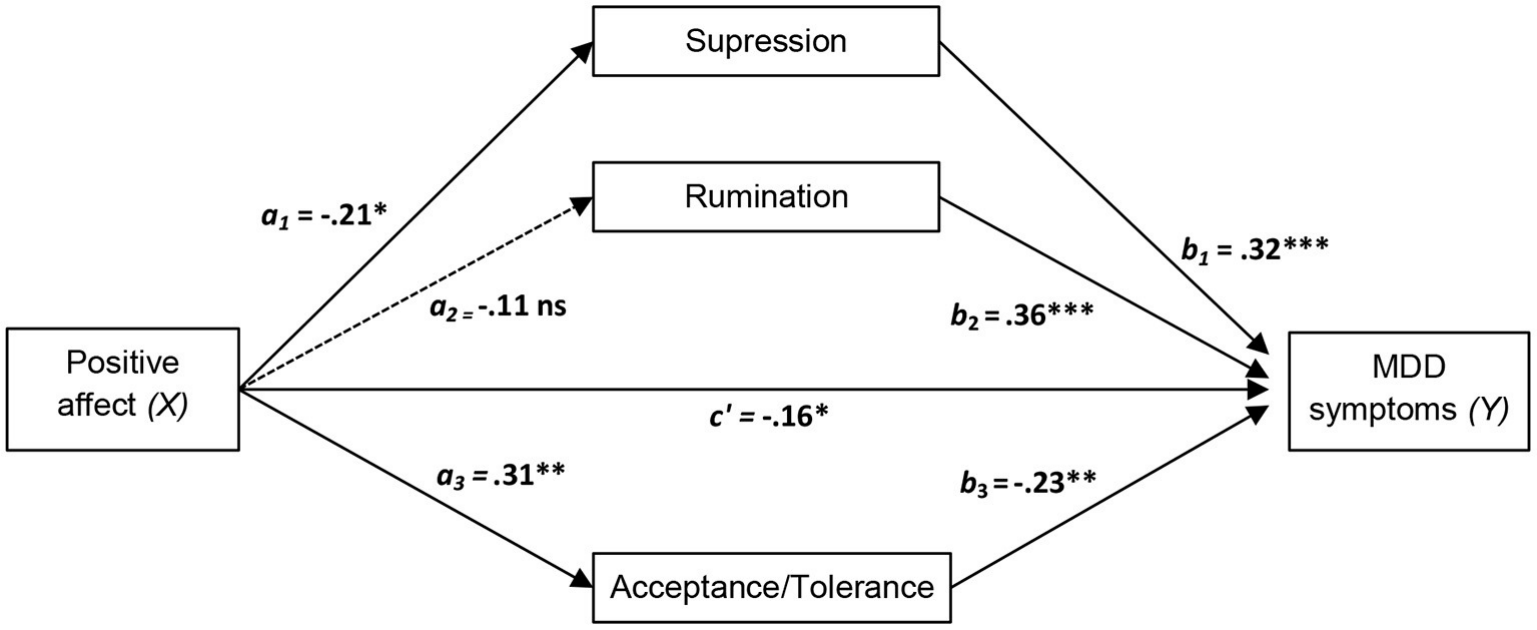
Diagram of the parallel multiple mediator model for the direct and indirect effects of positive affect on major depressive disorder (MDD) symptoms via emotion regulation strategies (acceptance/tolerance, suppression, and rumination). Fully standardized regression coefficients are shown. See Table 5 for indirect and total effect coefficients. *a*, effect of *X* on a mediator; *b*, effect of the mediator on *Y*; *c*′, direct effect of *X* on *Y*. *p < 0.05; **p < 0.01; ***p < 0.001.

**Figure 2:**
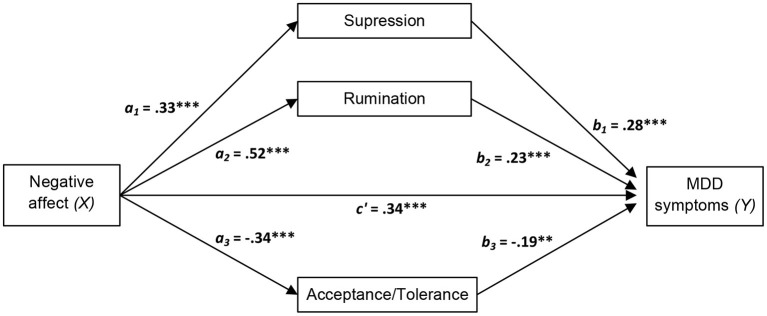
Diagram of the parallel multiple mediator model for the direct and indirect effects of negative affect on major depressive disorder (MDD) symptoms via emotion regulation strategies (acceptance/tolerance, suppression, and rumination). Fully standardized regression coefficients are shown. See Table 5 for indirect and total effect coefficients. *a*, effect of *X* on a mediator; *b*, effect of the mediator on *Y*; *c*′, direct effect of *X* on *Y*. **p < 0.01; ***p < 0.001.

**Figure 3:**
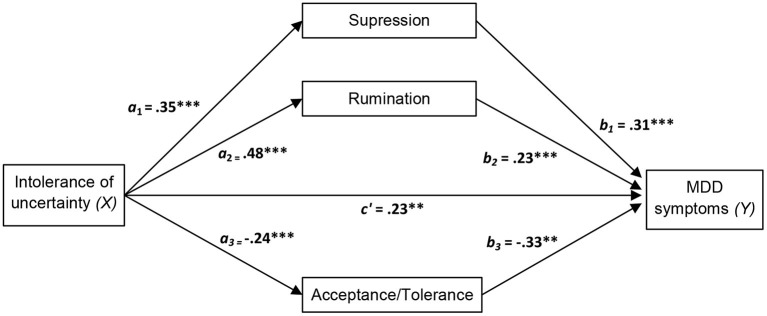
Diagram of the parallel multiple mediator model for the direct and indirect effects of intolerance of uncertainty on major depressive disorder (MDD) symptoms via emotion regulation strategies (acceptance/tolerance, suppression, and rumination). Fully standardized regression coefficients are shown. See Table 5 for indirect and total effect coefficients. *a*, effect of *X* on a mediator; *b*, effect of the mediator on *Y*; *c*′, direct effect of *X* on *Y*. **p < 0.01; ***p < 0.001.

In order to examine the possible role of emotion regulation strategies as mediators of the effect of higher level factors of the transdiagnostic model on MDD symptoms, we estimated the total, direct and indirect effects of positive affect, negative affect and intolerance of uncertainty on MDD symptoms through the three selected emotion regulation strategies (acceptance/tolerance, suppression and rumination), as these were the only emotion regulation variables that were statistically significant in the hierarchical multiple linear regression analysis after controlling for the remaining variables. Results of the mediation analyses are presented in Table 5 and Figures 1–3.

**Table 5:** Mediation of the effect of affectivity and intolerance of uncertainty on depressive disorder symptoms (RCADS-30-MDD) through emotion regulation strategies (acceptance/tolerance, suppression, and rumination) (fully standardized regression coefficients).

						**Specific indirect effect**								
			**Total indirect effect**			**Acceptance/tolerance**			**Suppression**			**Rumination**		
				**BC 95% CI**			**BC 95% CI**			**BC 95% CI**			**BC 95% CI**	
**MDD symptoms**	**Total effect**	**Direct effect**	**Coeff**.	**Lower**	**Upper**	**Coeff**.	**Lower**	**Upper**	**Coeff**.	**Lower**	**Upper**	**Coeff**.	**Lower**	**Upper**
Positive affect	−0.34^***^	−0.16^*^	−0.18^**^	−0.32	−0.04	−0.07^*^	−0.14	−0.02	−0.07^*^	−0.15	−0.01	−0.04**ns**	−0.12	0.03
Negative affect	0.62^***^	0.34^***^	0.28^***^	0.17	0.4	0.07^*^	0.01	0.15	0.09^**^	0.04	0.17	0.12^**^	0.05	0.23
Intolerance of uncertainty	0.53^***^	0.23^**^	0.30^**^	0.2	0.41	0.08^**^	0.02	0.15	0.11^**^	0.05	0.18	0.11^**^	0.04	0.19

*BC 95% CI, Bias–corrected 95% bootstrap confidence interval. RCADS-30, Revised Child Anxiety and Depression Scale−30; Coeff., regression coefficient; MDD, major depressive disorder. R^2^ for each model: 0.47 (positive affect), 0.53 (negative affect) and 0.49 (intolerance of uncertainty).*

* *p < 0.05;*

** *p < 0.01;*

*** *p < 0.001*.

As displayed in the figures, we hypothesized that negative affect and intolerance of uncertainty *(Xs)* lead to increased MDD symptom severity (*Y*) through a direct effect of *X* on *Y* and an indirect effect mediated by the three selected emotion regulation strategies (acceptance/tolerance, suppression and rumination). Accordingly, negative affect and intolerance of uncertainty should amplify MDD symptom severity directly and indirectly (through the activation of suppression and rumination and through the inhibition of acceptance/tolerance). An opposite pattern was predicted for positive affect, i.e., inhibition of rumination and suppression strategies and activation of acceptance/tolerance.

In line with the suggested pattern, significant direct and indirect effects were found for positive affect, negative affect, and intolerance of uncertainty. All effects of these variables were significantly mediated by acceptance/tolerance, suppression and rumination. As can be seen in Figures 1–3, all main standardized regression coefficients were statistically significant, except for the relationship between positive affect and rumination. Likewise, based on bootstrap confidence intervals, all three emotion regulation variables mediated the indirect effect of the predictors *(Xs)* on the outcome variable *(Y)*, except for rumination which did not mediate significantly the specific indirect effect of positive affect on MDD symptoms (see Table 5). The total effect of *X* on *Y* was significant for the three independent measures, although greater total effect sizes were found for negative affect (0.62, *p* < 0.001) and intolerance of uncertainty (0.53, *p* < 0.001) than for positive affect (−0.34, *p* < 0.001). This indicates, for example, that two people who differ by one unit in negative affect are estimated to differ by 0.62 in MMD symptoms (Sandín et al., 2015).

## Discussion

A main goal of the present study was to investigate the incremental validity of coronavirus fears in the prediction of anxiety and depressive disorder symptoms experienced during the COVID-19 pandemic crisis in a sample of adolescents, most of whom showed high levels of symptomatology. Results based on a series of hierarchical multiple regression analyses revealed that coronavirus fears explained additional variance in overall anxiety and depressive disorder symptoms (RCADS-30-Total score) and anxiety disorder symptoms (RCADS-30-Anxiety score), above the etiological factors of positive and negative affect, intolerance of uncertainty, distress tolerance and emotion regulation strategies. Coronavirus fears do not significantly predict specific depressive disorder symptoms (MMD subscale). These results indicate that coronavirus fears appear to be a relevant manifestation of the effect of the COVID-19 pandemic on adolescent mental health, and that such fears may significantly influence adolescent emotional health, increasing, for example, anxiety disorder symptoms. Findings also highlight the relevance of coronavirus fears as a consequence of the psychological impact of pandemic-related stress, as was initially suggested by Sandín et al. (2020c) and Lee et al. (2020). This is the first study to show that coronavirus fears can contribute to psychopathology in adolescents, and more specifically to the severity of anxiety disorder symptoms in this population.

A second aim of the present study was to examine the contribution of transdiagnostic vulnerability and protective factors to the prediction of the severity of anxiety disorder symptoms, major depressive disorder symptoms, and combined anxiety and depressive disorder symptoms. For this, three types of transdiagnostic constructs were selected, corresponding to each of the first three hierarchical levels of the transdiagnostic model of emotional disorders (Sandín et al., 2020a); that is to say, negative and positive affect (first level), intolerance of uncertainty and distress tolerance (second level), and emotion regulation strategies (awareness/understanding, acceptance/tolerance, reappraisal, self-instructions, suppression, rumination and distraction; third level). As expected, we found that predictors corresponding to each of the three levels of the transdiagnostic model (i.e., temperamental factors, clinical traits and coping strategies) account for a significant proportion of the variance in outcome variables. This is consistent with the model proposed by Sandín and colleagues, which assumes that factors of the three levels can uniquely contribute to the prediction of symptom severity.

However, not all transdiagnostic factors seem to be equally related to anxiety and depressive symptoms. Negative affect and intolerance of uncertainty appear to be powerful predictors of common symptoms of anxiety and depression, even after controlling for other vulnerability factors. This result is in line with our recent research findings that demonstrated a significant power of these variables to predict coronavirus fears during the lockdown in Spain (Sandín et al., 2020c). Likewise, some emotion regulation strategies, such as acceptance/tolerance, rumination and suppression were also significant predictors above the remaining predictors included in the hierarchical multiple regression models. As expected, adaptive strategies (acceptance and tolerance) were associated with less severity of anxiety and depressive symptoms, whereas maladaptive strategies (rumination and suppression) were associated with more severity. This finding suggests a possible role of emotion regulation strategies in amplifying or reducing symptoms of anxiety and depression, above positive and negative affect, intolerance of uncertainty, distress tolerance and coronavirus fears. Results of the present study are in accordance with data previously reported in the meta-analysis by Aldao et al. (2010). As reported these authors, anxiety and depression are closely related to certain maladaptive emotion regulation strategies, especially to rumination and avoidance. However, our findings also suggest the relevance of suppression, as well as some adaptive strategies (i.e., acceptance/tolerance). It is interesting to note that, in general, the pattern is similar for the three outcome variables, which provides important empirical support for the transdiagnostic model. The fact that some emotion regulation strategies (reappraisal, distraction, awareness/understanding) were less consistently associated with anxiety and depression symptoms is in line with the existing literature, suggesting that some strategies are more strongly related to emotional psychopathology than others (Aldao et al., 2010, 2016). Accordingly, these authors emphasized that the effect sizes for rumination and suppression were large and medium to large, respectively, whereas the effect size for reappraisal was small to medium. Overall, results indicate that, in addition to the well-known affective factors (positive and negative affect), intolerance of uncertainty and emotion regulation strategies function as transdiagnostic factors associated with the severity of anxiety and depression. These findings are in accordance with results of previous research on the transdiagnostic features of rumination (Ferrer et al., 2018) and intolerance of uncertainty (Pineda, 2018). Identifying which emotion regulation strategies play a greater role in the psychopathology of emotional disorders in general (i.e., as transdiagnostic strategies) or specific disorders in particular, is a topic of core interest for future research.

An unexpected result in the third regression analysis was the drop in predictive power of positive and negative affect and intolerance of uncertainty after emotion regulation strategies had been added to the model, when major depressive disorder symptoms (RCADS-30-MDD) were the outcome variable. We expected that the significant effect of positive affect, negative affect and intolerance of uncertainty would be maintained after adding the emotion regulation strategies to the model, as was the case when the outcome variables were RCADS-30-Total score and RCADS-30-Anxiety. Nevertheless, a possible explanation for this phenomenon could be that emotion regulation strategies mediate the effect of affectivity and intolerance of uncertainty on major depressive symptoms (RCADS-30-MDD).

Thus, an additional aim was to investigate the extent to which emotion regulation strategies mediate the effect of affectivity (positive and negative affect) and intolerance of uncertainty on depressive symptoms. We hypothesized that acceptance/tolerance, rumination and suppression each mediate the effect of affectivity and intolerance of uncertainty on depressive symptoms. We found that these three emotion regulation strategies significantly mediated the aforementioned relationships (the only exception was a non-significant association between positive affect and rumination). More specifically, significant indirect effects through acceptance/tolerance, rumination and suppression were found for the three relevant transdiagnostic factors (positive affect, negative affect and intolerance of uncertainty). Since significant direct effects were also found, emotion regulation strategies only partially mediate the effects of the three transdiagnostic factors on major depressive disorder symptoms. Thus, it can be concluded that affectivity and intolerance of uncertainty appear to influence adolescent depressive symptom severity, both in a direct and an indirect way. This result provides strong support for the transdiagnostic theory of emotional disorders (Barlow et al., 2004, 2014; Belloch, 2012; Sandín et al., 2012a; Ehrenreich-May et al., 2018), and more specifically for the hierarchical transdiagnostic model of emotional disorders (Sandín et al., 2020a).

The findings of the present study have several clinical implications. Given the fact that coronavirus fear was a unique predictor of the severity of overall internalizing symptoms, this variable could be managed by cognitive behavior therapy (CBT) programs aimed at the treatment of COVID-19-related anxiety symptomatology, since it is well-known that CBT is the evidence-based therapy of choice for the treatment of anxiety-based disorders (e.g., Moriana and Martínez, 2011). In addition, given the core role of transdiagnostic variables, including emotion regulation strategies (i.e., acceptance/tolerance, rumination, and suppression), transdiagnostic CBT (T-CBT) should be used to reduce negative affect, intolerance of uncertainty, and maladaptive emotion regulation strategies such as rumination and suppression, as well as to increase low levels of positive affect, tolerance and acceptance. Thus, understanding the unique contribution of transdiagnostic factors pertaining to the hierarchical transdiagnostic model of emotional disorders could help to implement T-CBT programs to target main transdiagnostic vulnerabilities. Recently, some transdiagnostic protocols have been designed for the prevention and treatment of emotional disorders in children and adolescents, especially of anxiety and depressive disorders (Ehrenreich-May et al., 2018; Sandín et al., 2019, 2020b; Orgilés et al., 2020a). Future research may want to prioritize these transdiagnostic variables in the corresponding modules of T-CBT programs. In addition, adolescents suffering from high levels of COVID-19-related anxiety and depression could benefit from transdiagnostic protocols delivered via internet (T-iCBT) (Sandín et al., 2020b; Fonseca and Osma, 2021). It has been suggested that internet-based interventions have several advantages compared with traditional face-to-face treatments, such as improved access to evidence-based treatments, a better cost-effectiveness and less stigma.

Some limitations of the present study should be noted. A first limitation is that common-method variance may have inflated the relationship between cognitive factors and self-reported anxiety and depression. Secondly, the sample size and the characteristics of the sample (a convenience sample) could limit the generalizability of the results. Thirdly, the cross-sectional nature of the study limits the conclusions that can be drawn concerning the etiological associations between the examined transdiagnostic factors and anxiety and depressive disorder symptoms. Thus, no causal inferences can be made between the variables included in the present study. Future longitudinal studies should assess transdiagnostic variables, coronavirus fears, anxiety and depression over time to allow for changes in anxiety and depressive symptom severity associated with changes in transdiagnostic processes and coronavirus fears. A fourth limitation of the present study was that it only focused on some transdiagnostic factors and three outcome measures of anxiety and depressive symptom severity. Future research would benefit from examining other transdiagnostic constructs and other combined measures of anxiety and depression. For example, anxiety sensitivity has been largely suggested as a main transdiagnostic factor implicated in the etiology of anxiety and depressive disorders.

## Data Availability Statement

The data are not publicly available due to restrictions (participants of this study did not agree for their data to be shared publicly, so supporting data is not available). Requests to access the datasets should be directed to Bonifacio Sandín, bsandin@psi.uned.es.

## Ethics Statement

The study was reviewed and approved by the Research Ethics Committee of the Universidad Nacional de Educaciistancia. Written informed consent to participate in this study was provided by the adolescents and their legal guardians (when the adolescent was under 16 years old).

## Author Contributions

BS, RV, and PC designed the study and wrote the paper. BS analyzed the data. VE, JGE, JS, and SA conducted the empirical study. JGE coordinated the empirical study. All authors contributed to manuscript revision, and read and approved the final version.

## Conflict of Interest

The authors declare that the research was conducted in the absence of any commercial or financial relationships that could be construed as a potential conflict of interest.

## Publisher's Note

All claims expressed in this article are solely those of the authors and do not necessarily represent those of their affiliated organizations, or those of the publisher, the editors and the reviewers. Any product that may be evaluated in this article, or claim that may be made by its manufacturer, is not guaranteed or endorsed by the publisher.
